# Portal vein thrombosis leading to pre-sinusoidal non-cirrhotic portal hypertension resulting in decreased synthetic function of the liver 

**Published:** 2019

**Authors:** Kevin Kline, Muhannad Al Hanayneh, Mohammad Bilal, Heather Stevenson-Lerner

**Affiliations:** 1 *Department of Internal Medicine, University of Texas Medical Branch, Galveston, TX, USA*; 2 *Division of Gastroenterology & Hepatology, University of Texas Medical Branch, Galveston, TX, USA*; 3 *Department of Pathology, University of Texas Medical Branch, Galveston, TX, USA *

**Keywords:** pathology, portal hypertension, gastrointestinal hemorrhage

## Abstract

Non-cirrhotic portal hypertension (NCPH), defined as elevated portal pressures in the absence of cirrhosis, is a relatively rare cause of elevated portal pressures in western countries. In NCPH decompensated liver disease is common, but complications are often mitigated by appropriate medical therapy. Liver synthetic function loss is uncommon. We present a unique case of a patient with biopsy proven NCPH, who eventually developed progressive loss of hepatic synthetic function in the setting of long standing portal hypertension. This loss of synthetic function corresponded with the interval development of incomplete septal cirrhosis (ISC), and progression of previously noted nodular regenerative hyperplasia in biopsies performed 7 years apart. Our patient’s clinical course was complicated by multiple hospitalizations for gastrointestinal hemorrhage. Patients with ISC have higher rates of bleeding varices when compared to patients with macronodular cirrhosis. While patients with NCPH typically have better overall survival and fewer bleeding complications than cirrhotic patients, this is typically attributed to the former having preserved synthetic function. It appears that the presence of ISC may be a poor prognosticator in patients with NCPH.

## Introduction

 Non-cirrhotic portal hypertension (NCPH) is a relatively rare cause of elevated portal pressures in western countries. NCPH can occur due to a varied group of causes, both known and idiopathic. They are defined collectively as elevated portal pressures in the absence of cirrhosis (1). NCPH typically results from a vascular lesion that leads to reduced portal venous flow (2). In NCPH decompensated liver disease is not uncommon, and often the disease initially presents as an episode of gastrointestinal bleeding. Complications of NCPH are often mitigated by appropriate medical therapy. In contrast to cirrhosis, synthetic function loss is uncommon (3, 4). We present a unique case of a patient with biopsy proven, pre-sinusoidal NCPH as a complication of portal vein thrombosis secondary to acute pancreatitis, who had multiple hospital admissions for upper gastrointestinal bleeding, and eventually developed progressive loss of hepatic synthetic function in the setting of long standing portal hypertension.

## Case Report

A 39 year-old incarcerated male with no prior medical history initially presented to our institution from another facility 11 years ago for an episode of necrotizing pancreatitis. The etiology of initial pancreatitis was unclear. 

Six months later, the patient returned to our hospital with abdominal pain. Computed tomography (CT) of the abdomen showed a large peripancreatic fluid collection and gallbladder sludge. He then underwent a cholecystectomy. 

**Figure 1:**
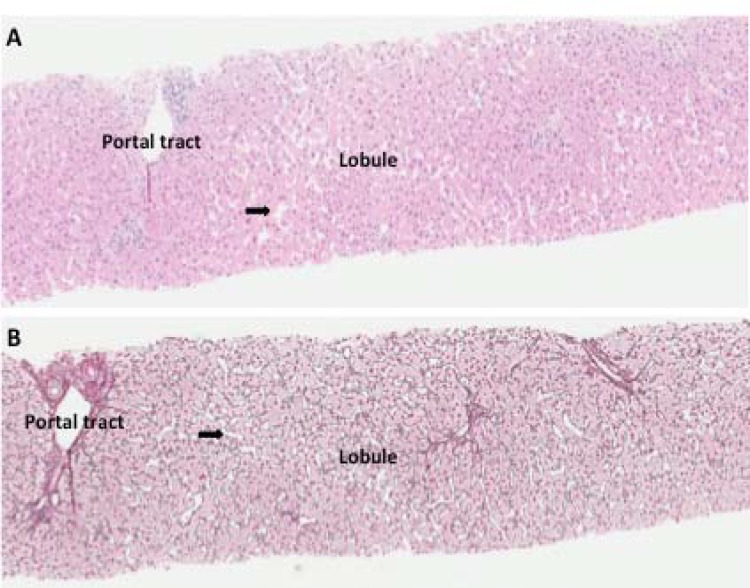
Histopathologic features observed in the first biopsy obtained from our patient. The biopsy showed primarily mild nodular regenerative hyperplasia-like changes with focal areas of sinusoidal dilatation. The portal tracts showed minimal inflammation and no other features to suggest developing non-cirrhotic portal hypertension. At this time point, there was minimal fibrosis (stage: 0/6). (A) H&E and (B) reticulin stain, which highlights the nodular regenerative hyperplasia changes. 10X objective

The patient presented to the hospital, one year after his index hospitalization, with ascites that was refractory to diuretics. Magnetic resonance imaging of the abdomen showed splenic vein thrombosis that extended into the portal venous system, not seen on his previous imaging studies. The patient had a peritoneal-venous shunt placed by surgery. A liver biopsy was performed demonstrating low-grade nodular regenerative hyperplasia (NRH) changes with mild perivenular sinusoidal dilatation, and mild portal fibrosis without evidence of bridging or chronic biliary obstruction (Figure 1). Workup for other common etiologies of hepatic injury including viral and autoimmune sources were negative. 

The patient was lost to follow-up for 7 years and eventually returned with melena. An esophagogastroduodenoscopy (EGD) conducted during that admission showed large esophageal varices in the distal esophagus and isolated gastric varices in the fundus of the stomach.

**Figure 2:**
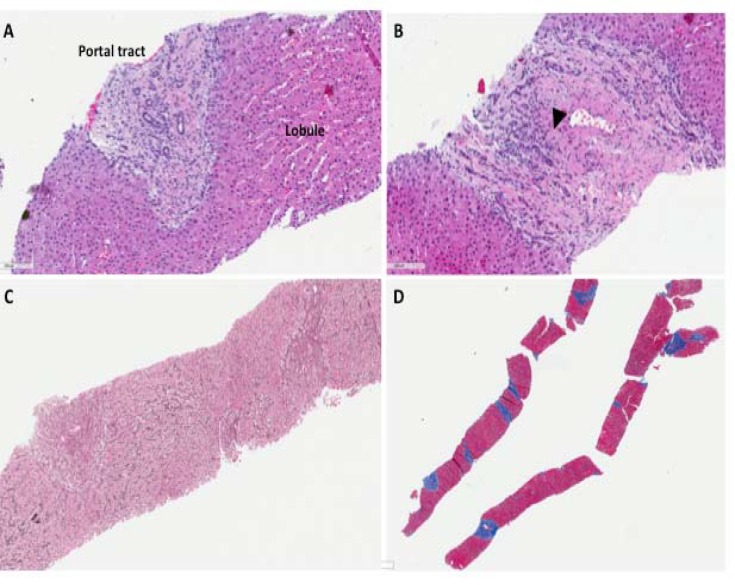
Histopathologic features observed in our patient’s liver biopsy 7 years later. Panels A and B show portal tracts with increased portal stromal sclerosis, absence and/or small portal veins with thickened vascular walls, and a moderate bile ductular reaction. The reticulin stain in panel C highlights the sunstantially increased nodular regenerative hyperplasia changes. At this later time point, there was increased portal sclerosis with absence of many of the portal veins and minimal periportal fibrosis with no bridging fibrosis was observed. Panels A and B: H&E at 20X objective; panel C: Reticulin stain at 4X objective, and panel D: Masson trichrome stain at 1X objective

Second liver biopsy was performed, demonstrating incomplete septal cirrhosis (ISC) and more advanced NRH (Figure 2). Corresponding with the progression of and increase in INR. At the time of his first biopsy, the patient maintained a serum albumin consistently between 3.5-4.5 g/dL. At the time of his subsequent biopsy, he demonstrated persistent hypoalbuminemia between 2.0-2.9 g/dL, despite ensuring adequate nutrition. His INR was initially in the range of 1.2-1.3, and eventually ranged from 1.6-1.9. At baseline, aspartate aminotransferase (AST) and alanine aminotransferase (ALT) remained within normal limits and stable between the biopsies. The alkaline phosphatase baseline increased over fourfold during this period (Table 1).

The patient was referred to transplant surgery, and selective splenic arterial embolization was recommended to reduce inflow to his gastric varicies. This procedure was performed by interventional radiology (IR), but only the proximal splenic artery could be embolized with collateral flow from the distal splenic, left gastric, dorsal pancreatic, and gastroepiploic branches, which reconstituted blood flow.

**Table 1:** Comparative view of baseline ranges of hepatic function at time of first biopsy and second biopsy demonstrating progressive loss of synthetic function

	Laboratory Normal Range	1^st^ Biopsy	2^nd^ Biopsy
INR (International Normalized Ratio)	<1.1	1.2-1.3	1.6-1.9
Albumin (g/dL)	3.5-5.0	3.5-4.5	2.0-2.9
AST (U/L)	13-40	25-34	26-36
ALT (U/L)	9-51	32-41	18-25
Alkaline Phosphatase (U/L)	34-122	87-120	391-568

**Figure 3:**
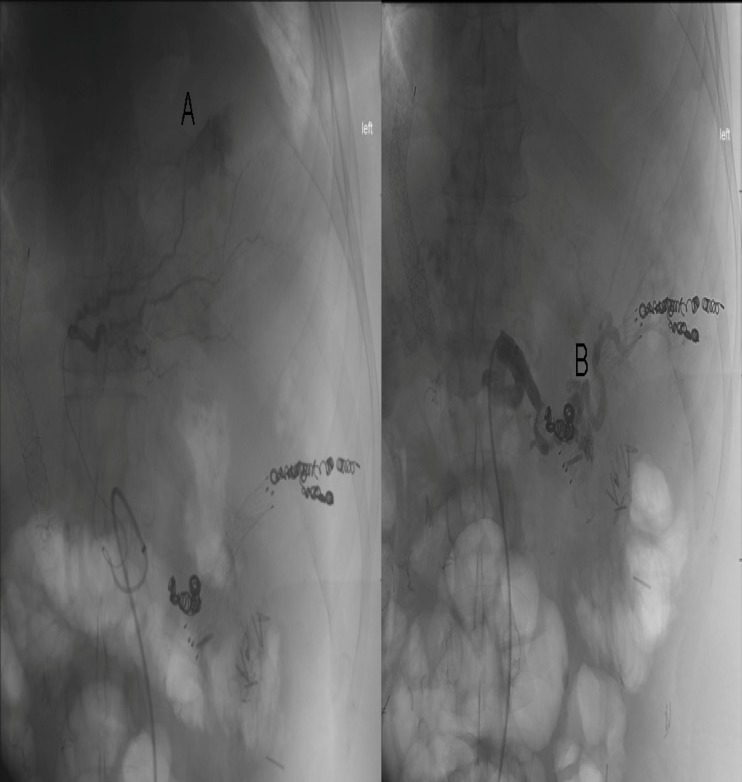
(A) Second branch of left hepatic artery supplying superior aspect of spleen prior to embolization (B) Dorsal pancreatic artery seen after embolization with retrograde flow of contrast, the coil of previous splenic artery embolization seen over the proximal portion of the vessel

He had subsequent admissions for melena and hematemesis. Eventually, a transjugular intrahepatic porto-systemic shunt and splenic vein recanalization with angioplasty and stent placement was performed by IR. Following this procedure, he did not have any overt gastrointestinal bleeding for six months, after which he presented with melena again; IR performed a visceral angiogram and venogram the left hepatic and dorsal pancreatic arteries were demonstrated to supply the spleen and were embolized (Figure 3). 

An attempt to catheterize the gastroepiploic artery was performed, but canalization was unsuccessful due to its tortuosity. The patient tolerated the partial splenic embolization with no significant subsequent bleeding, and was eventually discharged home. 

## Discussion

Portal vein thrombosis (PVT) is a rare but well recognized complication of pancreatic pathology (including acute pancreatitis and pancreatic cancer) (5). In patients with comorbid pancreatic disease and PVT, esophageal and gastric varices have been described at a rate of 90% and 36%, respectively (9). In patients with NCPH, current thought regarding the pathogenesis of the disease revolves around an initial “first hit” resulting in occlusions of small portal vein branches, causing a chronic ischemic state, with subsequent, associated histological changes. These changes can include NRH, and less often ISC (2). Our patient’s initial insult is believed to be his episode of acute pancreatitis, which resulted in splenic vein thrombosis and extension into the portal venous system. He then developed NRH, a common, early histopathologic change of NCPH (6,7). 

Decompensated liver disease in patients with NCPH has been described at rates of over 50% (3). In particular when looking at NRH, there may be a bias towards recognition of disease in patients with complications particularly as gastrointestinal bleeding is a common first presentation of disease (8). In a large autopsy study, NRH was seen in 2.6% of livers, but only 4.7% of those individuals had evidence of portal hypertension (8). Despite this, progressive loss of synthetic function is not typical in patients with NCPH (2,10). This progression corresponded with interval changes observed in his liver biopsies that were performed 7 years apart. While NRH is the most common histological abnormality seen in NCPH, ISC, a new finding in the second biopsy of our patient, is typically only seen in individuals with longstanding NCPH, and can be considered a bridge to true cirrhosis (2). While in pancreatic disease about 40% of adult cases there is histologic evidence of portal fibrosis, the progression of NRH and development of ISC has not been well documented in the literature and has primarily been described in patients who progress to liver failure (9, 11,12). Fiel *et al*. evaluated patients who received liver transplants with a previous biopsy diagnosis of hepatoportal sclerosis, a known cause of NCPH, 50% of which were found to have concurrent ISC, and seven out of eight had NRH. Of note, five of these eight cases were noted to have prolonged prothrombin times. The investigators hypothesized that synthetic dysfunction was secondary to significant hepatic parenchymal loss, as their gross specimens demonstrated shrunken livers. 

Our patient, while not in liver failure, did suffer significant complications from decompensated liver disease, manifested by diminished synthetic function. This occurred over a 7 year period. He was not on any hepatotoxic medications and denied any alcohol use. Important components in the management of NCPH include controlling episodes of acute gastrointestinal bleed, and preventing further episodes (13). Interestingly, patients with ISC were demonstrated to have higher rates of bleeding varices when compared to patients with macronodular cirrhosis (14). While patients with NCPH (including those with PVT from acute pancreatitis) typically have better overall survival and fewer bleeding complications than cirrhotic patients, this is typically attributed to the former having preserved synthetic function. While there is a relative paucity of data on the topic, it appears that the presence of ISC may be a poor prognosticator in patients with NCPH, and may have an association with synthetic function loss in this group of patients.

## Conflict of interests

The authors declare that they have no conflict of interest.
